# Treatment experiences for skin and soft tissue infections among participants of syringe service programs in North Carolina

**DOI:** 10.1186/s12954-021-00528-x

**Published:** 2021-07-30

**Authors:** Mary C. Figgatt, Zach R. Salazar, Louise Vincent, Diannee Carden-Glenn, Kelly Link, Lauren Kestner, Tyler Yates, Asher Schranz, Elizabeth Joniak-Grant, Nabarun Dasgupta

**Affiliations:** 1 Injury Prevention Research Center, University of North Carolina At Chapel Hill, 725 Martin Luther King Jr. Blvd, CB #7505, Chapel Hill, NC 27599 USA; 2 Department of Epidemiology, Gillings School of Global Public Health, University of North Carolina At Chapel Hill, 170 Rosenau Hall, CB #740, Chapel Hill, NC 27599 USA; 3 North Carolina Survivors Union, 1116 Grove Street, Greensboro, NC 27403 USA; 4 ekiM for Change, 620 Lynndale Ct, Suite C, Greenville, NC 27858 USA; 5 Community Hope Alliance, 2012 N Fayetteville St, Asheboro, NC 27203 USA; 6 Center for Prevention Services, 1117 E Morehead St #200, Charlotte, NC 28204 USA; 7 Guilford County Solution To the Opioid Problem, 1601 Walker Ave, Greensboro, NC 27403 USA; 8 Division of Infectious Disease, Department of Medicine, University of North Carolina At Chapel Hill, Chapel Hill, NC USA

**Keywords:** Drug use, Skin and soft tissue infections, Abscesses, Cellulitis, Endocarditis, Infectious disease, Syringe services programs, Syringe exchange programs, Harm reduction, Healthcare access, Wound care, Drug user led research

## Abstract

**Introduction:**

Bacterial and fungal infections, such as skin and soft tissue infections (SSTIs) and infective endocarditis (IE), are increasing among people who use drugs in the United States. Traditional healthcare settings can be inaccessible and unwelcoming to people who use drugs, leading to delays in getting necessary care. The objective of this study was to examine SSTI treatment experiences among people utilizing services from syringe services programs. This study was initiated by people with lived experience of drug use to improve quality of care.

**Methods:**

We conducted a cross-sectional survey among participants of five syringe services programs in North Carolina from July through September 2020. Surveys collected information on each participant’s history of SSTIs and IE, drug use and healthcare access characteristics, and SSTI treatment experiences. We examined participant characteristics using counts and percentages. We also examined associations between participant characteristics and SSTI history using binomial linear regression models.

**Results:**

Overall, 46% of participants reported an SSTI in the previous 12 months and 10% reported having IE in the previous 12 months. Those with a doctor they trusted with drug use-related concerns had 27 fewer (95% confidence interval = − 51.8, − 2.1) SSTIs per every 100 participants compared to those without a trusted doctor. Most participants with a SSTI history reported delaying (98%) or not seeking treatment (72%) for their infections. Concerns surrounding judgment or mistreatment by medical staff and self-treating the infection were common reasons for delaying or not seeking care. 13% of participants used antibiotics obtained from sources other than a medical provider to treat their most recent SSTI. Many participants suggested increased access to free antibiotics and on-site clinical care based at syringe service programs to improve treatment for SSTIs.

**Conclusions:**

Many participants had delayed or not received care for SSTIs due to poor healthcare experiences. However, having a trusted doctor was associated with fewer people with SSTIs. Improved access to non-judgmental healthcare for people who use drugs with SSTIs is needed. Expansion of syringe services program-based SSTI prevention and treatment programs is likely a necessary approach to improve outcomes among those with SSTI and IE.

## Introduction

Bacterial and fungal infections are increasing among people who use drugs (PWUD) in the United States [1–3]. These infections can range from common and typically not severe infections, such as skin and soft tissue infections (SSTIs), to infrequent but life-threatening infections, including infective endocarditis (IE), bone and spine infections [4]. SSTIs include abscesses and cellulitis, which are among the most commonly reported health problems by PWUD, particularly those who inject drugs. It has been estimated that over 50% of people who inject drugs have experienced SSTIs at some point in their lifetime [5, 6]. Often SSTIs can be self-resolving and localized bacterial infections. However, more virulent SSTIs result in serious systemic infections requiring hospitalization or surgery [5–8]. IE, a severe infection of the heart valves, has also increased substantially in recent years among PWUD [3, 9]. Unlike SSTIs, IE requires long hospital stays for treatment, frequently requires open heart surgery to replace infected valves, and is associated with high mortality rates [10, 11]. Both SSTIs and IE are commonly caused by *Staphylococcus aureus* [12–14] and have similar risk factors, including intravenous drug use without prior skin or hand washing, reusing or having limited access to new syringes, and injecting either intramuscularly or subcutaneously [5–7, 15].

PWUD face multiple barriers when accessing traditional medical services resulting in a long-standing history of mistrusting formalized medical care [16]. PWUD often cite mistreatment and stigma experienced in medical settings [17–21], sometimes noting that they are labeled as “drug seeking” and fear experiencing withdrawal if hospitalized [21]. As a result, some PWUD turn to treatment options outside traditional healthcare settings, for example self-treating abscesses and obtaining antibiotics from sources other than healthcare providers [21–23]. In contrast, community-and peer-based harm reduction organizations, such as syringe services programs (SSPs), have earned the trust of PWUD, and thus are uniquely situated to offer education and services to address SSTIs and IE [24, 25].

The objective of this study was to describe and quantify personal and healthcare experiences with, as well as treatment strategies for, SSTIs and IE among PWUD utilizing SSP services. It also aimed to understand the reasons PWUD have for alternative treatment strategies and how SSPs might better meet the needs of PWUD with SSTIs.

## Methods

### Study design

This cross-sectional health needs assessment was initiated by the SSPs in response to increasing concerns about SSTIs among the populations served by SSPs. To recruit survey sites, staff from one SSP (North Carolina Survivors Union) contacted other SSPs using a statewide contact list. Among approximately 30 SSPs in North Carolina at the time of the survey, four additional programs agreed to participate, resulting in a total of five survey sites.

Surveys were administered from July through September 2020. During this time, the coronavirus pandemic (COVID-19) precautions resulted in reduced SSP operations for some sites and shorter durations of engagements with SSP participants, although sites reported that demands for services had increased since pandemic onset. We used a convenience sampling approach to identify survey participants. Specifically, SSP staff approached persons seeking services (e.g., those obtaining safe drug use supplies or naloxone) and asked about their interest in participating in a short survey. Surveys were self-administered at the SSP via electronic tablets using a standardized instrument [26]. The median time to complete the survey was approximately 8 minutes. Any person who provided consent and completed the survey questions for the key variables of interest (i.e., history of SSTIs or IE) was included in the analysis. 95% of participants completed the survey while the remaining participants partially completed the survey. Only two participants completed less than half of the survey questions. Participants with missing values to specific questions were not included in the results for those specific questions. People with lived experience of drug use and representatives of SSPs were involved in the study conceptualization and design, including survey development.

### Measures

All survey measures were self-reported by survey participants. The full survey is available at https://doi.org/10.17615/4g3h-j073. Demographic characteristics included age, gender, race, and ethnicity. Healthcare access measures included health insurance coverage and access to a trusted doctor for drug use-related concerns. SSTI and IE history was defined by those events occurring ever in the participant’s lifetime or within the previous 12 months. To improve the accuracy of questions relating to SSTI history, the survey tool asked participants whether they would be willing to see photos of several types of SSTIs to aid recall. Based on this response, participants were either shown these photos in the survey tool prior to the SSTI history questions or automatically proceeded to these questions without displaying the photos.

Participants were asked about injection of specific drugs, including cocaine, crack-cocaine, fentanyl, heroin, methamphetamine, and prescription opioids, in their lifetime and during the 12 months prior to survey administration. Participants were also asked about whether they had missed a shot (i.e., unintentional subcutaneous or intramuscular injection) or skin popped (i.e., intentional subcutaneous injection) in the previous 12 months. Participants were asked about SSTI treatment experiences including treatment strategies (including oral antibiotics, the source from which the oral antibiotics were obtained, and types and sources of treatment) and barriers to treatment (including healthcare experiences and accessibility). Participants were also asked to provide suggestions to improve their SSTI treatment experiences.

### Statistical analysis

We examined frequencies and percentages of the study population’s overall drug use and healthcare access characteristics by SSTI and IE history. We used unadjusted and adjusted binomial linear regression models to estimate prevalence differences and corresponding 95% confidence intervals for potential predictors (drug use characteristics and healthcare access) of SSTI history within the previous 12 months. Participants with missing values for any of the specific variables included in each model were excluded (i.e., complete case analysis). Prevalence differences can be interpreted as the excess or reduced number of people with SSTIs per every 100 people when comparing one group to another (e.g., those who had recently injected cocaine compared to those who had not). Adjusted models included indicator variables for self-reported race (dichotomized as white versus non-white because of small sample size for non-white categories), gender (dichotomized as men versus not men because non-binary identities were queried but had small sample size), and health insurance coverage (dichotomized as yes or no). Adjusted models evaluating health insurance as a potential predictor only included indicator variables for race and gender. All statistical analyses were conducted using SAS 9.4 (Cary, North Carolina).

### Ethical considerations & participation of people with lived experience of drug use

Among those approached at the SSPs, individuals who displayed an interest in completing the survey were provided with a brief description of the survey purpose and intended use of the data. After reviewing this information, these individuals were asked if they would still be willing to participate. No compensation was provided. This study was reviewed and approved by the University of North Carolina at Chapel Hill Institutional Review Board. People with lived experience of drug use were involved in survey conceptualization and design, questionnaire development, data collection, and interpretation of results.

## Results

### Participant characteristics

Of the 281 people approached, 37% (n = 105) agreed to participate in the survey. Response proportion varied from 17 to 71% by site, with each site contributing between 11 and 36 participants. Of the 105 participants, 51% (n = 52) were men and 87% (n = 89) were white. The median age of participants was 39 years-old (range: 22–69, missing n = 31). A total of 34% (n = 35) of participants had health insurance coverage and 29% (n = 30) had a doctor they trusted with health concerns relating to drug use (Table 1). 93% (n = 96) had injected any type of drugs in the previous 12 months, which could have included intravenous, intramuscular, or subcutaneous injection. The most common drugs injected in the previous 12 months were heroin (80%, n = 77), fentanyl (72%, n = 69), methamphetamine (62%, n = 59), and cocaine (59%, n = 57).

**Table 1:** Skin and soft tissue infection (SSTI) and infective endocarditis (IE) history during the previous 12 months, drug use characteristics during the previous 12 months, and healthcare access among survey participants

	All Participants		Participants with a History of SSTIs During the Previous 12 Months		Participants with a History of IE During the Previous 12 Months	
	N = 105	Percent (%)	N = 47	Percent (%)	N = 10	Percent (%)
*SSTI & IE history during the previous 12 months*						
SSTI history	47	46.1	–	–	6	60.0
* Missing*	3				0	
IE history	10	10.0	6	12.8	–	–
* Missing*	5		0			
*Drug use characteristics during the previous 12 months*						
Drugs injected						
Benzodiazepines	12	12.5	7	14.9	1	10.0
Cocaine	57	59.4	31	66.0	4	40.0
Crack	35	36.5	22	46.8	4	40.0
Fentanyl	69	71.9	36	76.6	6	60.0
Heroin	77	80.2	38	80.9	6	60.0
Methamphetamine	59	61.5	30	63.8	6	60.0
Opana ER	8	8.3	5	10.6	0	0.0
Oxycontin OP	9	9.4	5	10.6	0	0.0
Other pain pills	7	7.3	5	10.6	1	10.0
* Missing*	*9*		0		0	
Injected drugs	96	93.2	46	97.9	10	100.0
*Missing*	2		0		0	
Skin popped	32	32.0	18	38.3	2	20.0
* Missing*	5		0		0	
Missed a shot	70	70.0	37	78.7	7	70.0
* Missing*	5		0		0	
*Healthcare access*						
Health insurance coverage	35	34.3	14	29.8	1	10.0
* Missing*	3		0		0	
Has a doctor who is trusted with concerns related to drug use	30	29.4	9	19.2	1	10.0
* Missing*	3		0		0	

### SSTI and IE prevalence

Overall, 64% (n = 65) of participants had a lifetime history of SSTIs and 46% (n = 47) had a history of SSTI in the previous 12 months. Among those with a lifetime history of SSTIs, most reported one (38%, n = 18) or multiple SSTIs (two to four: 45%, n = 21) in the previous 12 months. Seventeen percent (n = 17) of participants had been told by a doctor that they had IE in their lifetime and 10% (n = 10) were told this in the previous 12 months. Of the 17 respondents with a lifetime history of IE, eight (47%) had surgery for the infection and five (29%) reported they were living with untreated IE. The proportion of participants with a history of SSTI in the previous 12 months varied by survey site, from as low as 35% (n = 12) at one site to as high as 92% (n = 11) at another site.

### Predictors of SSTI and IE

Adjusted models accounted for gender, race, and insurance status. Cocaine injection was associated with an increase in recent SSTI relative to other substances injected: There were 17 more people with SSTI per every 100 participants (95% CI = − 2.0, 37.0) who injected cocaine compared to those who had not injected cocaine in the previous 12 months (Table 2). Crack-cocaine injection (24 more people with SSTI per 100 every participants, 95% CI = 3.5, 43.5) and fentanyl injection (19 more people with SSTI per every 100 participants, 95% CI = − 3.6, 40.7) were also independently associated with an increased prevalence of recent SSTI. Missing shots was associated with 18 more people with SSTIs per every 100 participants (95% CI = − 3.9, 39.7) in the previous 12 months.

**Table 2:** Unadjusted and adjusted prevalence differences between participant characteristics and skin and soft tissue infection (SSTI) history during the previous 12 months

	N with SSTI/Total Participants	SSTI Prevalence Difference (N per 100 persons)	SSTI Prevalence Difference 95% Confidence Interval
*Drug use characteristics during the previous 12 months*			
Cocaine injection			
Unadjusted estimate	47 102	18.8	(− 0.2, 37.9)
Adjusted estimate	47/100	17.5	(− 2.0, 37.0)
Crack-cocaine injection			
Unadjusted estimate	47/102	25.5	(5.8, 45.3)
Adjusted estimate	47/100	23.5	(3.5, 43.5)
Fentanyl injection			
Unadjusted estimate	47/102	18.8	(− 1.1, 38.8)
Adjusted estimate	47/100	18.6	(− 3.6, 40.7)
Heroin injection			
Unadjusted estimate	47 102	13.4	(− 8.5, 35.2)
Adjusted estimate	47/100	12.6	(− 14.0, 39.1)
Methamphetamine injection			
Unadjusted estimate	47/102	11.3	(− 8.1, 30.7)
Adjusted estimate	47/100	8.4	(− 11.4, 28.2)
Skin popping			
Unadjusted estimate	47/100	13.6	(− 7.2, 34.4)
Adjusted estimate	47/100	12.9	(− 8.3, 34.0)
Missed a shot			
Unadjusted estimate	47/100	19.5	(− 1.0, 40.0)
Adjusted estimate	47/100	17.9	(− 3.9, 39.7)
*Healthcare access*			
Health insurance coverage			
Unadjusted estimate	47/100	− 8.8	(− 29.3, 11.7)
Adjusted estimate	47/100	− 10.3	(− 31.3, 10.7)
Access to trusted doctor			
Unadjusted estimate	47/100	− 24.3	(− 44.4, − 4.2)
Adjusted estimate	47/100	− 26.9	(− 51.8, − 2.1)

Prevalence differences reflect the difference in the prevalence of SSTI history in the previous 12 months among those with the drug use or healthcare access characteristic versus those without the characteristic of interest, excluding those with missing values. For example, the prevalence difference for cocaine injection compared the prevalence of SSTI history in the previous 12 months among those who injected cocaine versus those who had not in the previous 12 months. Adjustment variables included race, gender, and health insurance coverage for all predictors, excluding health insurance coverage. Health insurance coverage was adjusted for race and gender

Having a trusted doctor for drug use-related concerns was protective for recent SSTI. There were 27 fewer people with a recent SSTI per every 100 participants (95% CI = 2.1, 51.8) with access to a doctor they trusted compared to those without access to a doctor, after adjusting for gender, race, and health insurance status.

### Treatment strategies and barriers to treatment

Among the 65 participants with a lifetime history of SSTIs, 71% (n = 46) reported having a SSTI that did not resolve on its own. In order to treat a SSTI, 63% (n = 39) reported taking antibiotic pills that were not prescribed to them.

Of those experiencing a SSTI that did not resolve on its own, nearly everyone reported delaying (98%, n = 45) or not receiving treatment (72%, n = 33) for their SSTI. Participants cited judgment or mistreatment from medical staff (54%, n = 25), self-treatment (52%, n = 24), time commitments (37%, n = 17), legal concerns (30%, n = 14), and previous bad experiences when getting treatment (28%, n = 13) as main reasons for delaying or not receiving treatment (Table 3).

**Table 3:** Main reasons participants had delayed or not sought care for a SSTI (N = 46)

	Number of Participants	Percent of Participants, %
Judgement or mistreatment by medical staff	25	54.3
Self-treatment	24	52.2
Too busy or it takes too much time	17	37.0
Legal concerns (e.g., warrants, child protective services)	14	30.4
Bad experiences when sought care other times	13	28.3
Costs too much	7	15.2
Wanting to avoid withdrawal from being in the hospital	6	13.0
Transportation barriers	6	13.0
Thought it would go away on its own	6	13.0
Afraid of finding out the infection is serious	5	10.9
Worried about the medical procedures (e.g., drainage)	3	6.5

### Treatment experiences for most recent SSTI

Among the 65 participants with a lifetime history of SSTI, we examined the experiences of the 55 whose infection was resolved at the time of survey. For these participants, seeking help soon after noting the infection was common; 70% (n = 38) had sought help from doctors or friends within a few days to a few weeks after first noting the infection. The most common sources of treatment included healthcare providers (62%, n = 34), and friends or family members (49%, n = 27) (Table 4). 71% (n = 39) of participants had infections that required an emergency room visit, 62% (n = 34) were given intravenous antibiotics, 39% (n = 21) were hospitalized overnight, and 24% (n = 13) had surgery for their infection. Most of their infection resolved after either: a) an incision followed by drainage of the infection and after taking antibiotics (46%, n = 25), or b) after drainage without antibiotic treatment (36%, n = 20). 86% (n = 47) took oral antibiotics to treat their infection; of which, 87% (n = 41) were prescribed by a healthcare provider and 13% (n = 6) were obtained from friends and family. Treatment adherence was high: among those prescribed antibiotic pills, 78% (n = 29) reported finishing the full course of antibiotics.

**Table 4:** Treatment characteristics for participant’s most recent skin or soft tissue infection (SSTI) among those with a history of SSTIs who no longer had the infection at the time of survey administration (N = 55)

	**Number of Participants**	**Percent, %**
*Sources of SSTI treatment*		
Dealer or supplier	2	3.6
Friend or family member	27	49.1
Harm reduction organization	3	5.5
Healthcare provider (e.g., doctor's office, hospital)	34	61.8
*Infection required:*		
Emergency room visit	39	70.9
Hospitalization	21	38.9
Intravenous antibiotics	34	61.8
Antibiotic pills	47	85.5
Surgery	13	23.6
*Infection resolved:*		
On its own	6	10.9
Following antibiotics treatment without drainage	4	7.3
Following drainage but without antibiotic treatment	20	36.4
Following drainage and antibiotic treatment	25	45.5

### Suggestions to improve SSTI treatment

When asked about strategies to improve their SSTI treatment experiences, many participants suggested increased access to free antibiotics and clinics. Some suggested these services be based at SSPs. Select responses relating to access based at SSPs included: *“Free antibiotics”*, *“Have a place like a clinic for treatment without being judged or mistreated to go for help”*, and *“Have a nurse or doctor come to the exchange.”* Some participants also reported barriers relating to health insurance and mistreatment such as, *“Most people won’t go to [the] doctor because of no insurance or cost. Provide free treatment”* and *“Insurance is a huge barrier. Mistreatment due to being a drug user. Better treatment by healthcare professionals.”*

## Discussion

We found a substantial burden of SSTIs and IE in a convenience sample of PWUD seeking services from SSPs in North Carolina. Participants who had a doctor they trusted with drug use-related concerns had a lower prevalence of recent SSTI compared to those without a trusted doctor. However, over two-thirds of participants did not have a trusted doctor. Commonly reported reasons for delaying or not receiving SSTI treatment included stigma, past mistreatment by medical staff, and legal concerns (e.g., warrants, child protective services). Delaying or not receiving treatment was also related to practical concerns, such as time constraints, financial cost, and transportation, all of which impacted accessibility to clinical care. In addition, many participants sought treatment from sources outside of traditional healthcare settings, such as the use of antibiotics obtained from sources other than healthcare providers and treatment from friends and family.

The burden and severity of SSTIs and IE underscores the need for treatment and prevention innovation. These two infections are closely related. 60% of our participants with a history of IE in the previous 12 months also had a history of SSTIs in the previous 12 months. Prevention of SSTIs, such as harm reduction education and access to safe drug use supplies, may also lead to an individual’s subsequent reduced risk of IE. Stigma and mistreatment by medical staff are common barriers to care for PWUD [18–20]. Respectful and judgment free patient-provider relationships are urgently needed to improve SSTI outcomes among PWUD. Such respectful treatment empowers PWUD to make more informed decisions regarding their health and wellness. PWUD may turn to people they have trusted relationships with for care, including friends and family. This could occur in the form of obtaining antibiotics or having support while attending wound care appointments. SSPs often build trust with PWUD and may serve as an ideal setting for SSTI treatment services. For example, wound care has been successfully implemented at some SSPs in the United States, but is not universal [27–29]. Ginoza et al. reported success with a medical student-led wound care clinic that provides low-barrier SSTI care and linkages to care for other resources, such as food and housing [30]. Such programs may serve as a model for other community-based clinics. Given that most PWUD in this study did not have health insurance, community-based SSTI care should consider offering no or minimal cost services, such as on-site clinical care, wound care kits, and education about safe self-care of wounds.

64% of participants in our survey had a lifetime history of SSTIs. Comparable studies have found similarly high prevalence estimates [5, 6, 8]. Previous studies have also found injection of specific types of stimulants to be associated with increased SSTI occurrence [5, 31–35]. In line with this, we found cocaine and crack-cocaine injection were associated with an increased prevalence of recent SSTI history. Yet, fentanyl injection was also associated with an increased prevalence of recent SSTI history. We did not obtain information, however, on whether cocaine, crack-cocaine, and fentanyl were used simultaneously or independently; therefore, it is not clear how these results differ from previous associations with speed balling (i.e., mixing heroin and cocaine). However, similar associations were not observed with heroin and methamphetamine injection.

In line with our results, other research has also identified that some people obtain antibiotics from sources other than healthcare providers to treat SSTIs; however, recent research has been qualitative and recent estimates of this type of antibiotic use are not established [21, 23]. For their most recent SSTI or ever in their lifetime, 13% and 63% of participants, respectively, had taken antibiotics pills that were not prescribed to them in order to treat a SSTI. This suggests a need for expanded access to antibiotics with clinical guidance and, more generally, expanded access to SSTI care.

This study has several notable strengths. Our study team included people with lived experience of drug use who were involved in conceptualization and design, survey development, and results interpretation. Specifically, the initiation of this needs assessment by people with lived experience of drug use led to a timely and impactful topic with relevant and culturally appropriate survey questions. Our study team’s structure provided a platform to quickly contextualize results and translate how the results can inform practice and advocacy efforts. This study also included five SSPs from different regions of North Carolina, representing a wide range of PWUD populations and SSPs. In addition, much of the existing research surrounding SSTI treatment among PWUD has focused on self-treatment strategies. Our study adds to this previous research by identifying specific barriers to clinical treatment and incorporating PWUD recommendations to improve SSTI treatment.

Some limitations should be noted. First, these results are not intended to be generalizable to all PWUD seeking services; selection bias arising from non-participation and convenience sampling could not be avoided while complying with COVID-19 social distancing mandates. In addition, survey participation varied by SSP which may be due to the type of site (e.g., fixed versus mobile locations). While stigma and privacy concerns could be drivers of non-participation, we believe this was minimized due to SSP staff administering the surveys as opposed to outside researchers. In addition, we were unable to provide incentives for study participation. Future studies should consider providing incentives to be respectful of participants’ time and increase participation. Second, we did not collect information on the participant injection frequency, which is likely a major predictor of SSTIs and other injection-related injury and infections [36]. Third, the study sample size resulted in low precision of our estimates of SSTI predictors. The study also lacked sufficient sample size to examine predictors of IE. In addition, IE is associated with high mortality rates. Therefore, our study would not include PWUD who died for IE, potentially underestimating the true impact of IE on this population. However, this is one of the first SSP-based surveys to examine treatment barriers in the context of IE. Additional research should examine drug use characteristics and healthcare access predictors of these infections among a larger population of PWUD. Fourth, our survey relied on self-reported history of SSTI and IE. Misclassification is possible, particularly depending in timing of infection in relation to data collection and infection severity. Lastly, our study did not establish temporality of events nor other factors that likely contribute to SSTI occurrence. Therefore, measures of association should not be interpreted causally, but rather as potential predictors of disease.

## Conclusions

Improved access to quality, non-judgmental healthcare is needed for PWUD with SSTIs. Future research should examine which specific treatment approaches are beneficial, preferred, and well-accepted by PWUD. Additionally, education about SSTI prevention should be, and often is, routinely offered at SSPs. Similar harm reduction education should be offered in healthcare settings that serve PWUD with SSTIs or PWUD at risk of developing SSTIs. Additional resource allocation, such as funding for wound care programs and SSP-based clinical services, is likely an important strategy to prevent these infections and improve outcomes among PWUD with SSTIs and IE.

## Data Availability

The survey used in this study is available at https://doi.org/10.17615/4g3h-j073. The datasets during and/or analyzed during the current health needs assessment are available from the corresponding author on reasonable request.

## Abbreviations

SSTI: Skin and soft tissue infection
IE: Infective endocarditis
PWUD: People who use drugs
SSP: Syringe service program (
